# Two Groups of *Thellungiella salsuginea* RAVs Exhibit Distinct Responses and Sensitivity to Salt and ABA in Transgenic *Arabidopsis*

**DOI:** 10.1371/journal.pone.0153517

**Published:** 2016-04-19

**Authors:** Shaohui Yang, Cui Luo, Yingjin Song, Jiehua Wang

**Affiliations:** School of Environmental Science and Engineering, Tianjin University, Tianjin, 300072, China; Institute of Genetics and Developmental Biology, Chinese Academy of Sciences, CHINA

## Abstract

Containing both AP2 domain and B3 domain, RAV (Related to ABI3/VP1) transcription factors are involved in diverse functions in higher plants. A total of eight *TsRAV* genes were isolated from the genome of *Thellungiella salsuginea* and could be divided into two groups (A- and B-group) based on their sequence similarity. The mRNA abundance of all *Thellungiella salsuginea TsRAVs* followed a gradual decline during seed germination. In *Thellungiella salsuginea* seedling, transcripts of *TsRAVs* in the group A (*A-TsRAVs*) were gradually and moderately reduced by salt treatment but rapidly and severely repressed by ABA treatment. In comparison, with a barely detectable constitutive expression, the transcriptional level of *TsRAVs* in the group B (*B-TsRAVs*) exhibited a moderate induction in cotyledons when confronted with ABA. We then produced the “gain-of-function” transgenic *Arabidopsis* plants for each *TsRAV* gene and found that only *35S*:*A-TsRAVs* showed weak growth retardation including reduced root elongation, suggesting their roles in negatively controlling plant growth. Under normal conditions, the germination process of all *TsRAVs* overexpressing transgenic seeds was inhibited with a stronger effect observed in *35S*:*A-TsRAVs* seeds than in *35S*:*B-TsRAVs* seeds. With the presence of NaCl, seed germination and seedling root elongation of all plants including wild type and *35S*:*TsRAVs* plants were retarded and a more severe inhibition occurred to the *35S*:*A-TsRAV* transgenic plants. ABA treatment only negatively affected the germination rates of *35S*:*A-TsRAV* transgenic seeds but not those of *35S*:*B-TsRAV* transgenic seeds. All *35S*:*TsRAVs* transgenic plants showed a similar degree of reduction in root growth compared with untreated seedlings in the presence of ABA. Furthermore, the cotyledon greening/expansion was more severely inhibited *35S*:*A-TsRAVs* than in *35S*:*B-TsRAVs* seedlings. Upon water deficiency, with a wider opening of stomata, *35S*:*A-TsRAVs* plants experienced a faster transpirational water loss than wild type and *35S*:*B-TsRAVs* lines. Taken together, our results suggest that two groups of TsRAVs perform distinct regulating roles during plant growth and abiotic defense including drought and salt, and A-TsRAVs are more likely than B-TsRAVs to act as negative regulators in the above-mentioned biological processes.

## Introduction

The RAV (Related to ABI3/VP1) family represents a unique group of plant-specific transcription factors since some members within this family contain two DNA-binding domains, e.g. a Basic 3 (B3) domain and an Apetala2 (AP2) domain. First identified in the *viviparous1* (*vp1*) mutant from *Zea mays* and the *ABA insensitive-3* (*abi3*) mutant from *Arabidopsis*, the B3 domain has been shown to be involved in the process of seed maturation and ABA regulation of gene expression [1,2,3]. The AP2 domain could be found within many AP2/ERF family members and has been implicated in plant hormone signal transduction and responses to biotic, pathogenic, and environmental cues.

In *Arabidopsis*, there are thirteen RAV family members [4], six of which contain both B3 domain and AP2 domain [5,6]. So far, only AtRAV1 and AtRAV2 have been functionally investigated in several aspects of plant physiology and development. Transgenic *Arabidopsis* plants overexpressing *AtRAV1* or *AtRAV2* were reported to result in growth-retarded phenotypes including reduced lateral root, smaller rosette leaf and slightly late flowering [4,7]. Transgenic tobacco lines constitutively expressing a soybean *RAV2* ortholog also exhibited slower plant growth rate, reduced root elongation and delayed flowering, especially under short day conditions [8]. In contrast, *rav1* and *rav2* single mutants showed slightly promoted growth patterns in the early stage of development [4]. Considering these results, RAV1 and RAV2 have been believed to act as negative regulators during plant growth and floral transition.

*RAV* genes have also been shown to be induced by various biotic and abiotic environmental stimuli including pathogen infection, salicylic acid, osmotic stress, cold, high salinity, wounding and exogenous hormone application [9,10,11,12,13]. However, contradictory results including the repressed expression of *AtRAV1* and *RAV2* by drought, salt and ABA treatment have also been reported [4]. Thus, whether or not and how RAV transcription factors are involved in the adaptive strategies of stressed plants still remains unclear. It is well known that both drought and high salinity can trigger the production of ABA, and drought-inducible genes could also be activated by salt-stress and ABA. In literature, *Arabidopsis rav1* mutant has been shown to exhibit an ABA insensitivity to root growth inhibition, and conversely overexpression of *AtRAV1* or *AtRAV2* in transgenic *Arabidopsis* resulted in an increased sensitivity to ABA inhibition of seed germination and root growth [14]. However, in another work published recently, *AtRAV1* overexpressing and T-DNA insertion mutant lines have been described as insensitive and hypersensitive, respectively, to ABA inhibition of root growth [15]. In addition to the above-mentioned discrepancies in gene expression profiles and induction patterns, results of functional analyses were not always consistent either. For example, transgenic cotton (*Gossypium hirsutum*) expressing *AtRAV1* and *2* showed improved resistance to drought and water usage efficiency [16]. Overexpression of a pepper *RAV1* was reported to enhance the tolerance to NaCl [10] and transgenic tomato overexpressing its endogenous *SlRAV2* also resulted in tolerance to bacterial wilt [17]. In contrast to these promising results, overexpression of *GhRAV1* in *Arabidopsis* resulted in transgenic plants sensitive to high salinity, drought and exogenous ABA instead [18]. *AtRAVs* were also recently demonstrated to play negative roles in drought and salt tolerance in an ABA-independent manner [4]. So far, there is no clear explanation for these experimental discrepancies. In addition, all functional analyses performed on *RAVs* to date have been focused on *RAV1*, *RAV2* and their homologues, and little information exists in literature about the expression patterns and function roles of other *RAV* gene family members.

In order to make a systematic investigation on the responses of RAV family members to salt and ABA, and analyze their respective contribution to salt and drought tolerance, we isolated all eight RAV subfamily members that contain both AP2 and B3 domains from *T*. *salsuginea*, a close relative of *Arabidopsis* with a exceptionally high resistance to cold, drought, salt and oxidative stresses [19,20,21,22]. Compared to *A*. *thaliana*, a modest expansion of the RAV family occurred to the genome of *T*. *salsuginea*, which might be associated with the adaptive strategies of *T*. *salsuginea* to its extreme environment. Interestingly, two groups of ThRAVs separated by their characteristic sequence features also exhibited distinctively different induction patterns when confronted with salt and ABA treatment. When introduced into *Arabidopsis*, overexpression of *A-TsRAVs* and *B-TsRAVs* resulted in various responses towards abiotic stresses including NaCl, water loss and ABA treatment, implying their different regulatory roles during plant growth and development.

## Materials and Methods

### Plant materials, growing conditions and stress treatments

Seeds of *T*. *salsuginea* were obtained from Dr. Yinxin Li at the Institute of Botany of the Chinese Academy of Sciences. To investigate the responses of *TsRAVs* to NaCl and ABA, seeds of *T*. *salsuginea* were germinated on 1/2 MS medium and grown vertically on plates for 15 days. Upon stress treatments, seedlings were first acclimated in 1/2 liquid medium for half an hour before being treated by 200 mM NaCl or 5 *μ*M ABA. Rosette leaves and roots were separately sampled from three biological replicates at 0, 0.5, 1, 3, 6, 12, and 24 h time points.

### Sequence isolation and analysis

Sequence alignments of TsRAV and AtRAV proteins were performed with Clustal W [23] using default parameters. A phylogenetic tree was constructed with MEGA 5.0 [24] using the neighbor-joining method and the tree reliability was set to 1000 bootstrap replicates. The identification of B3 and AP2 domains was based on the genome annotation and verified by the InterProScan and Pfam online tools with defaulted parameters [25,26,27]. Motif identification within protein sequences was carried out using the MEME Suite tool version 4.11.0 [28] with the following parameters: optimum width of 5–50 amino acids, any number of repetitions of a motif, and the maximum number of motifs set at 30 with a *E*-value<4.6e-5 and a *P*-value<1.1e-5.

### Gene isolation, expression profile analyses and construction of transgenic plants

Total RNA of *T*. *s*alsuginea seedling was extracted using the EasyPure Plant RNA Kit (TransGen, China) and cDNA synthesis was performed using the EasyScript® First-Strand cDNA Synthesis SuperMix (TransGen, China) with the Oligo(dT)_18_ reverse primer. Amplifications of *actin* were used as controls for the semi-quantitative PCR analysis. Quantitative Real-Time PCR (qRT-PCR) analysis of cDNA was performed on a PikoReal 96 Real-time Thermal Cycler and PikoReal Software (V2.2) (Thermo Fisher Scientific, Finland) using Real Master Mix SYBR Green I (NEWBIO, China). The following thermal cycle conditions were used: 95°C for 2 min, followed by 45 cycles of 95°C for 20 s and 58°C for 20 s, 72°C for 30 s. All reactions were performed in triplicate from three independent pooled samples (50 plants per sample). Relative quantification of specific mRNA levels was analyzed using the cycle threshold (Ct) 2^-ΔΔCt^ method. Relative expression levels are normalized using the housekeeping gene *actin* and shown in folds of the expression value in untreated samples. The sequences of all primers used in this study are listed in S1 Table.

To generate *Arabidopsis* transgenic lines overexpressing *TsRAVs*, the cDNA fragment of each *TsRAV* was cloned into the pK2GW7I expression vector under the control of *CaMV 35S* promoter. The plasmids were introduced into *Agrobacterium tumefaciens* strain individually to transform *Arabidopsis thaliana* ecotype Columbia (Col-0) using the floral dip method [29]. The kanamycin-resistant seedlings were transplanted into soil and homozygous T3 seeds were used for the following transgenic analyses.

### Stress and hormonal treatments on transgenic plants

For seed germination under abiotic stresses, transgenic T3 seeds were surface-sterilized and germinated on 1/2 MS agar medium containing 100 mM NaCl or 1 *μ*M ABA for 7 days at 22°C with a 16 h-light/8 h-dark cycle. Fifty seeds per replicate were examined with three replicates for each treatment. For measurement of root growth, *Arabidopsis* seeds were germinated on 1/2 MS agar medium for 5 days and the seedlings were transferred to fresh medium containing 200 mM NaCl or 30 *μ*M ABA, and plates were placed vertically on shelves to facilitate comparison of root growth. To determine the drought tolerance in a quantitative manner, leaves were detached from each plant and placed in Petri dishes that were kept at 40% relative humidity in a growth chamber, and the loss of fresh weight was determined at the indicated time points. Stomatal aperture measurements were performed on epidermis from leaves of 4-week-old plants and values are the means for at least 50 apertures from at least three experiments.

## Results and Discussion

### Identification and classification of *T*. *salsuginea* RAV family

**Fig 1:**
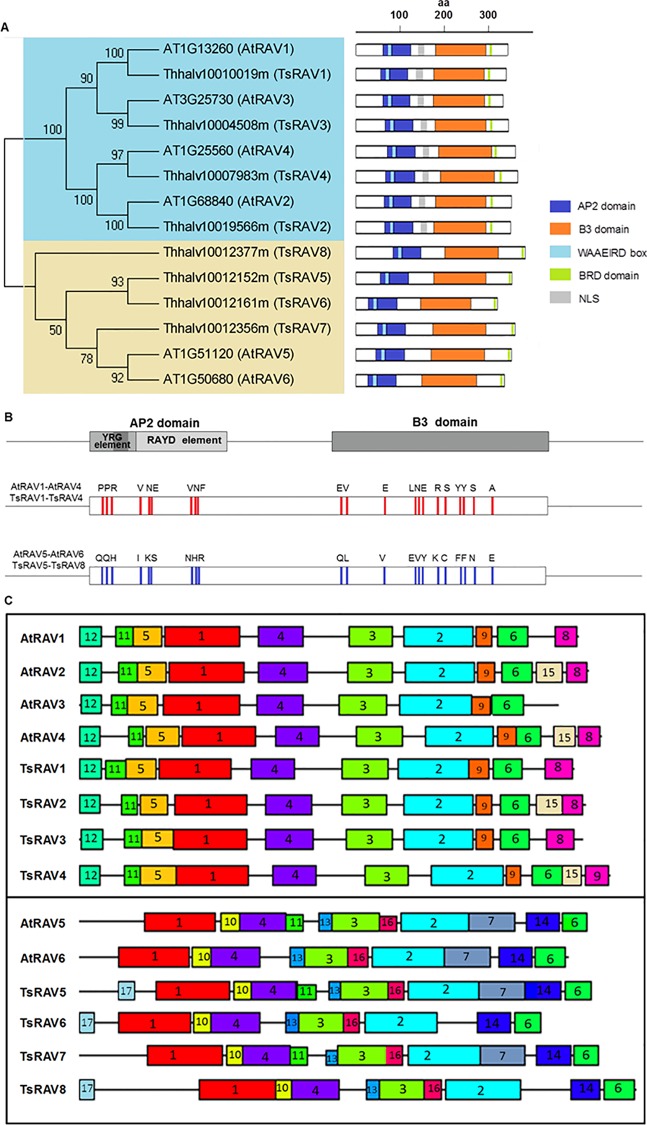
Sequence characterization of RAV family members of *Thellungiella salsuginea* and *Arabidopsis thaliana*. (A) Phylogenetic tree of the RAV family members in *Thellungiella salsuginea* and *Arabidopsis thaliana*. The phylogenetic tree was constructed using full-length protein sequences by the maximum-likelihood method with MEGA 5.0 and a bootstrap value of 1,000. The two major phylogenetic clades are designated as groups A and B. Shown on the right are diagrams of RAV proteins with information on the structure and position of different protein domains. (B) RAV subfamily-specific amino acids and their locations along the RAV full-length sequences. The amino acid sequences in boxes represent the conserved AP2 and B3 DNA-binding domains, which are characteristic of RAV transcription factors. The locations of the conserved YRG and RAYD elements are indicated as well. (C) Schematic illustrations of the types and distributions of motifs for each TsRAV family members with a same group. Motifs were identified using the MEME search tool and numerically marked according to their statistical significance (low *E*-value) in a descending order.

The *Arabidopsis* genome contains six RAVs that contain both AP2 domain and B3 domain [30] and AtRAVs share 35–79% amino acid identity throughout their full-length sequences. Using RAV sequences from *Arabidopsis* and other species as query sequences to search the *T*. *salsuginea* genome database, a total of 8 *TsRAVs* were isolated with the predicted full-length proteins ranging from 320 to 384 amino acids (S2 Table). In the phylogenetic tree, eight TsRAVs and six AtRAVs obviously split into two groups (Fig 1A). The group A includes TsRAV1-4 and AtRAV1 (At1g13260), AtRAV2/TEM2 (AT1g68840), AtRAV3/RAV1L (At3g25730) and AtEDF1/TEM1 (AT1g25560), and the group B includes TsRAV5-8 and two AtRAVs (At1g51120 and At1g50680) (Fig 1A). At their N-terminal regions, all eight TsRAVs contain an AP2 DNA-binding domain that recognizes a consensus CAACA sequence [31] and includes the conserved YRG and RAYD elements as well as a conserved 7-aa WAAEIRD box motif [32] (Fig 1A). At the C-terminal regions, all eight TsRAVs contain a B3 domain that recognizes a consensus CACCTG sequence and a 15-aa B3 repression domain (BRD) (GNSKTLRLFGVNMEC) that is responsible for the repressive activity in many B3 super-family members (Fig 1A). A short peptide composed of five amino acid residues, R/KLFGV, has been demonstrated to be crucial to maintain the repressive activity of the BRD domain [33]. In this work, TsRAVs in group A and group B include a RLFGV and M/KLFGV core sequence, respectively, which was also identified in rice RAV homologues [33]. The function of RAVs as transcriptional repressors has been demonstrated in several plant species. For example, TEM1 and GmRAV can repress flowering by binding to and repressing the promoters of *FT* genes in *Arabidopsis* and soybean, respectively [34,35]. Notably, each A-TsRAV but not a single B-TsRAV contains a nuclear localization sequence, which might contribute significantly to their roles as transcriptional regulators (Fig 1A). In addition to the BRD domain, a close comparison of the amino acid sequences between A-TsRAVs and B-TsRAVs revealed that they are characterized by distinctly different amino acids at some highly conserved positions and these group-specific residues might in turn affect the secondary structure of RAV proteins as well as their DNA-binding activities (Fig 1B). We also analyzed the motif composition of AtRAVs and TsRAVs using the MEME program. In total, 17 conserved motifs were detected and some of them appeared to be highly divergent between two groups (Fig 1C). For example, motifs 5 and 12 located in front of the AP2 domain, motif 9 within the B3 domain and motif 8 at the C-end of the RAV protein are highly conserved in the group A-TsRAVs, but are absent in the B-group RAVs. On the contrary, motif 10 within the AP2 domain, motifs 13 and 16 within the B3 domain and motif 14 located in front of the BRD domain are present in all group B-TsRAVs but missing in the group A-TsRAVs (Fig 1C).

### Transcriptional change of *TsRAVs* during seed germination and their responses to salt and ABA application

**Fig 2:**
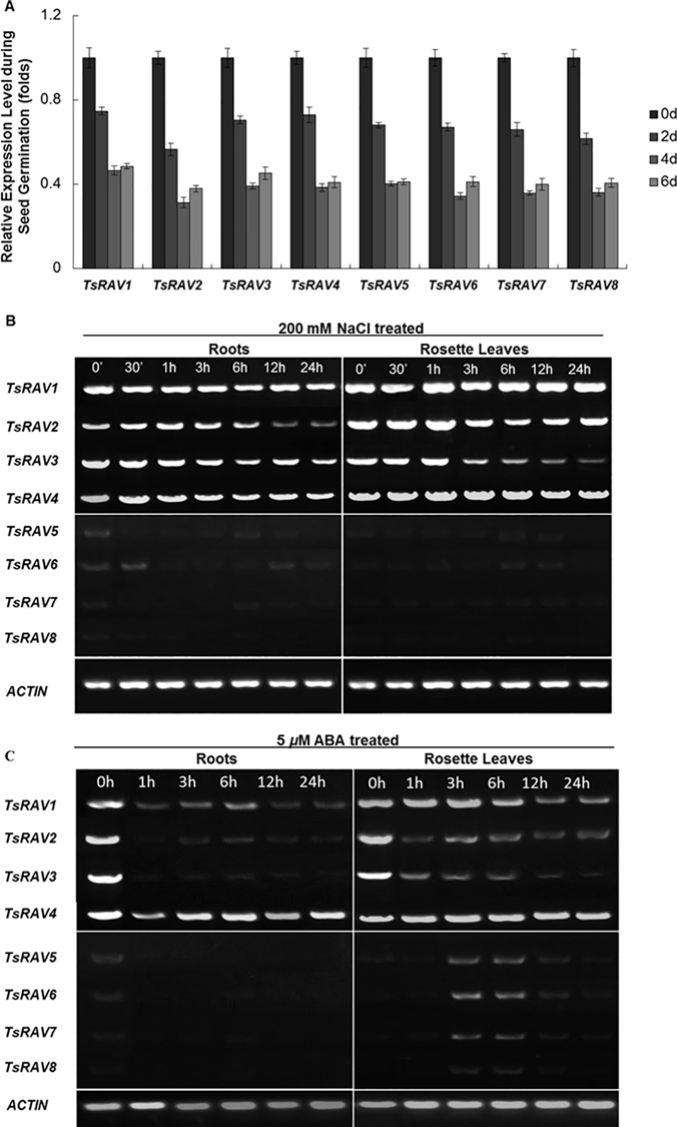
Expression of *TsRAV* genes during seed germination and in response to salt and ABA treatments. (A) qRT-PCR assay of *TsRAVs* transcription in *Thellungiella salsuginea* during seed germination. Analyses were performed on seeds at 0, 2, 4, and 6 DAI (days after imbibition). The transcription levels of genes were quantified relative to that of *actin*. Each data bar represents the means ± SE (Standard Error) of three replicates. (B) Semi-quantitative RT-PCR assay of *TsRAVs* transcription in *Thellungiella salsuginea* seedlings upon 200 mM NaCl treatment. Total RNA was extracted at various time intervals from leaves and roots of 15-d-old seedlings. The cDNA samples are normalized using an *actin* gene as an internal control. (C) Semi-quantitative RT-PCR assay of *TsRAVs* transcription in *Thellungiella salsuginea* seedlings upon 5 *μ*M ABA treatment. Total RNA was extracted at various time intervals from leaves and roots of 15-d-old seedlings. The cDNA samples are normalized using an *actin* gene as an internal control.

We then determined the transcript level of eight *TsRAV* genes during *Thellungiella salsuginea* seed germination. It has been reported that in *Arabidopsis*, the expression level of *AtRAV1* was very low in imbibed seed and was dramatically increased during seed germination, and its down-regulation could slowed down seed germination [15]. However, in this work, within six days after imbibition, the transcripts of all *TsRAVs* experienced a persistent and significant decrease until the end of the germination process, indicating that TsRAVs are negatively involved in seed germination process (Fig 2A).

We then analyzed the transcriptional changes of all *TsRAVs* in response to salt and ABA in seedling rosette leaves and roots. First, when treated by 200 mM NaCl, similar responses were observed in these two types of tissues for all eight *TsRAV* genes (Fig 2B). In the group A, *TsRAV1-4* exhibited a high constitutive expression level under normal conditions. Upon NaCl stress, *TsRAV1* and *4* remained a stable expression level within 24 hours, while *TsRAV2* and *3* showed a gradual and moderate decrease in their expression level (Fig 2B). In the group B, *TsRAV5-8* only accumulated a detectable yet low level of transcripts normally and did not significantly respond to the presence of NaCl (Fig 2B). In literature, the expression level of *AtRAV1* and *AtRAV2* has been reported to be up-regulated by various external and environmental cues including low temperature, darkness, wounding, drought, salt, and pathogen attack [10,11,36]. *RAV1* orthologs from *Brassica napus*, pepper and cotton have also been reported to be inducible by NaCl [10,13,18]. However, the expression of *AtRAV1* and *2* were recently shown to be repressed by drought and salt [4] just as described in this work. A possible explanation for these discrepancies might be the internal developmental regulations of *RAV* genes [37] and/or related to the time points and tissue types that were collected for analyses. Although *TsRAVs* were not found to be inducible by salt in this study, we could not rule out the possibility of them to play a role in response to salt stress.

ABA is a plant hormone known for its involvement in various signal transduction pathways and exogenous ABA is capable of inhibiting seed germination and early seedling growth [38,39]. In a previous work, the expression of *AtRAV1-3* were shown to be repressed significantly by ABA treatment for 24 hours [4]. In this work, upon the treatment of 5 *μ*M ABA, *A-TsRAVs* and *B-TsRAVs* responded to ABA in a distinctly different manner and only *A-TsRAVs* but not *B-TsRAVs* were transcriptionally repressed (Fig 2C). *TsRAV1* showed a moderate and continuous decline in its expression level and the transcript level of *TsRAV4* kept fairly stable, and the responses of both genes in shoot and root were similar (Fig 2C). For *TsRAV2* and *TsRAV3*, their transcript abundances in roots was rapidly repressed by ABA within one hour and then disappeared drastically thereafter; while in rosette leaves, their transcripts followed a gradual decrease (Fig 2C), indicating the differential responses of two tissues to ABA application. For *B-TsRAVs*, despite that their expression level kept fairly low in root regardless of the presence of ABA, they could be transiently but obviously triggered by ABA in leaves with a transcriptional peak observed at 3 h and 6 h (Fig 2C). Thus, when exposed to NaCl or ABA, the only significant induction observed in the current work happened to *B-TsRAVs* in the ABA-treated leaves (Fig 2B and 2C). Under normal or salty conditions, *B-TsRAVs* always maintained a low expression level while *A-TsRAVs* showed a high level of constitutive expression and were repressed to various extents by salt or ABA application. In summary, although that the elevated expression of *RAV1* by ABA has previously been reported in cotton and pepper [10,18], our results were consistent with the transcriptional repression of *AtRAV1* by exogenous ABA [15]. Meanwhile, we revealed that upon abiotic stress treatment, *TsRAVs* could respond in a significantly different manner in various types of plant tissues, which might be one of the factors contributing to the discrepancy of different experimental systems.

### Effects of *TsRAVs* overexpression on *Arabidopsis* seed germination and early seedling growth with or without NaCl

**Fig 3:**
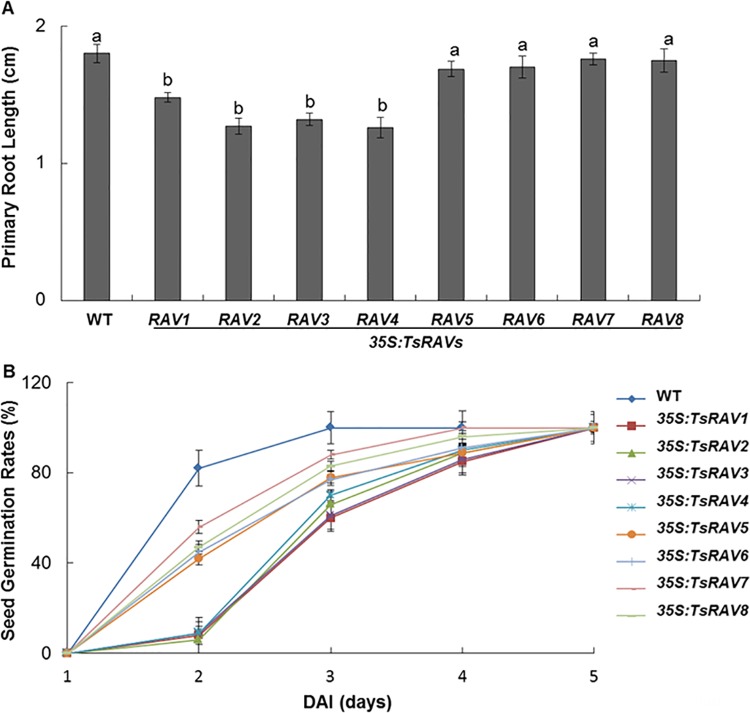
Phenotypic characterization of *35S*:*TsRAVs* transgenic *Arabidopsis* plants under normal conditions. (A) Primary root length of 7-d-old *35S*:*TsRAVs* transgenic *Arabidopsis* seedlings grown on 1/2 MS media. Each data bar represents the means ± SE of three replicates. More than 50 seedlings were measured for each replicates. Different letters indicate significant differences among means (*P*<0.05 by Tukey’s test). (B) Germination rates of *35S*:*TsRAVs* transgenic *Arabidopsis* seeds during a 5-day period on normal 1/2 MS media. Each data bar represents the means ± SE of three replicates. More than 100 seeds were measured in each replicated.

To understand the involvement of *TsRAV* genes in response to stressed conditions, we generated the “gain-of-function” transgenic lines by introducing each of the eight *TsRAV* genes individually into *Arabidopsis* under the control of the *CaMV 35S* promoter. For each *TsRAV* transgene, three homozygous overexpressing (OE) lines with the highest transgene expression level were selected for further analysis (S1 Fig). The previously reported inhibitory effects of AtRAV1 and AtRAV2 on plant growth [7,34,40,41] supported their roles as negative regulators during *Arabidopsis* growth and development [1]. In this work, we observed severe root growth retardation in *35S*:*A-TsRAVs* transgenic lines but not in *35S*:*B-TsRAVs* lines (Fig 3A). The only morphological defect detected in the vegetative and floral tissues was the narrower and longer leaves shown by *35S*:*TsRAV7* transgenic plants (S2 Fig).

We then compared the germination rates of the *TsRAVs*-overexpressing transgenic seeds with that of wild type seeds. With wild type seeds basically completed germination within 2 days after imbibition (DAI), transgenic seeds overexpressing *TsRAVs* were inhibited in germination process to different extents, and the germination of *A-TsRAVs* transgenic seeds was much more repressed than that of *B-TsRAVs* transgenic seeds (Fig 3B). This difference was most significant at DAG 2 with the average germination rate of *A-TsRAV* transgenic lines as 8% compared to that of *B-TsRAVs* as 47% and that of WT as 82% (Fig 3B). Previously, the *RAV1*-overexpressing transgenic *Arabidopsis* have been reported to show normal seed germination pattern compared with wild type in the absence of salt [4], whereas our results indicated that even without any stress, the germination rates of *TsRAVs* overexpressing lines have already been reduced significantly.

**Fig 4:**
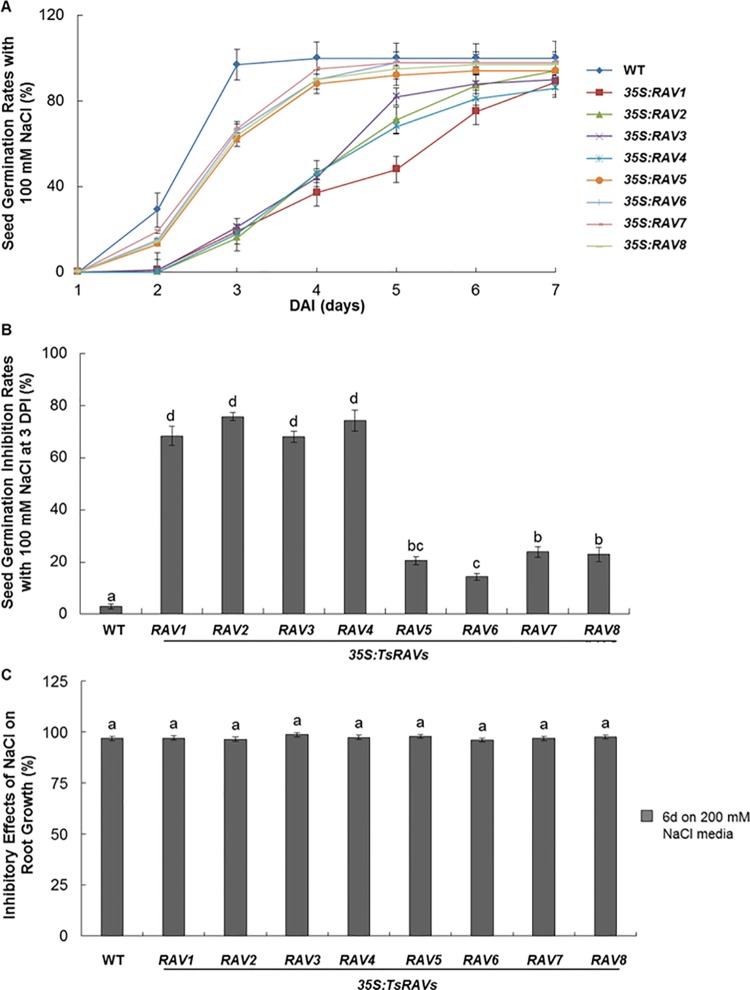
NaCl sensitivity of *35S*:*TsRAVs* transgenic *Arabidopsis* plants. (A) Germination rates of *35S*:*TsRAVs* transgenic *Arabidopsis* seeds on 1/2 MS media with 100 mM NaCl. Each data bar represents the means ± SE of three replicates. More than 100 seeds were measured in each replicate. (B) Inhibitory effect of 100 mM NaCl on *35S*:*TsRAVs* transgenic *Arabidopsis* seed germination rates. Each data bar represents the mean ± SE of three replicates. More than 50 seedlings were measured in each replicate. Different letters indicate significant differences among means (*P*<0.05 by Tukey’s test). (C) Inhibitory effect of 200 mM NaCl on *35S*:*TsRAVs* transgenic *Arabidopsis* seedling root elongation. Seedlings were grown on normal media for 5 days before being transferred onto 1/2 MS medium with 200 mM NaCl and grown for other 6 days. Each data bar represents the mean ± SE of three replicates. More than 50 seedlings were measured in each replicate. Different letters indicate significant differences among means (*P*<0.05 by Tukey’s test)

Salts at high concentration could inhibit the germination of *Arabidopsis* and ABA plays a role in this process [42]. When wild type and transgenic seeds were germinated on media containing 50, 75 or 100 mM NaCl, all types of *TsRAVs* transgenic seeds experienced a considerable reduction in their germination rates (S3 Fig and Fig 4). However, the inhibitory effect of *A-TsRAVs* overexpression on seed germination was much more significant than that of *B-TsRAVs* overexpression (S3 Fig and Fig 4). At DPI 3 when nearly all WT seeds were germinated on the salt-containing media, the germination rates of *B-TsRAVs* and *A-TsRAVs* transgenic seeds reached 60% and 20%, respectively (Fig 4A and 4B), indicating that as negative regulators of seed germination process, *A-TsRAVs* were much more competent than *B-TsRAVs*. A previous work by Li and co-workers also reported that the inhibition effect of NaCl on the germination of *RAV* transgenic seeds was greater than on that of wild type seeds, although that they did not detect any significant difference in their germination rates on normal 1/2 MS medium [18]. In summary, our data showed that overexpression of *RAV* genes did not enhance the salt tolerance of transgenic plants at the seed germination stage, which was contrary to the report that pepper *RAV1* improved salt tolerance when overexpressed in transgenic *Arabidopsis* lines [10].

We then determined whether or not *TsRAVs*-overexpressing seedlings have improved salt tolerance during early developmental stage by transferring 5-day-old seedlings from normal 1/2 MS media to NaCl-containing media. As described earlier, under normal conditions, *35S*:*A-TsRAVs* lines had shorter root length whereas *35S*:*B-TsRAVs* lines had similar root length as WT. After transferring to medium supplemented with 200 mM NaCl, both WT and *TsRAVs* OE lines were inhibited by about 96% in their primary root length (S4 Fig and Fig 4C). However, when the inhibitory effect of NaCl on root elongation was calculated against their respective performance on normal media, it could be seen that all *TsRAVs* transgenic plants responded to NaCl to a similar extent (Fig 4C), indicating overexpression of *A-TsRAVs* and *B-TsRAVs* in *Arabidopsis* did not change the sensitivity of transgenic plants to salt.

### Effects on germination and early seedling growth of *TsRAVs*-transgenic *Arabidopsis* with or without ABA

**Fig 5:**
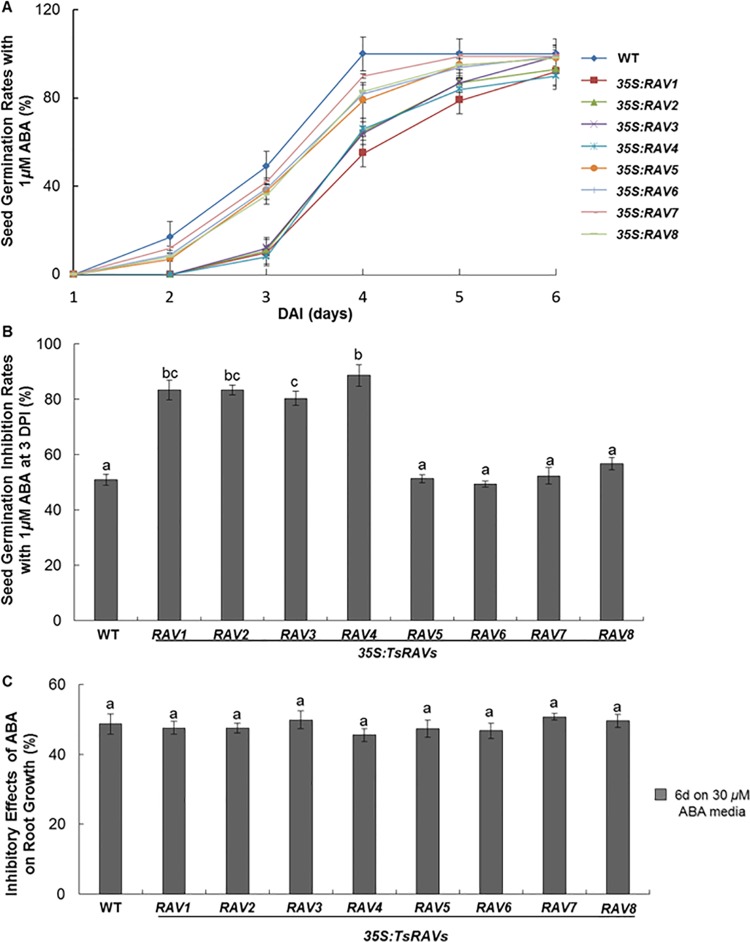
ABA sensitivity of *35S*:*TsRAVs* transgenic *Arabidopsis* plants. (A) Germination rates of *35S*:*TsRAVs* transgenic *Arabidopsis* seeds on 1/2 MS media with 1 *μ*M ABA. Each data bar represents the means ± SE of three replicates. More than 100 seeds were measured in each replicate. (B) Inhibitory effect of 1 *μ*M ABA on *35S*:*TsRAVs* transgenic *Arabidopsis* seed germination rates. Each data bar represents the mean ± SE of three replicates. More than 50 seedlings were measured in each replicate. Different letters indicate significant differences among means (*P*<0.05 by Tukey’s test). (C) Inhibitory effect of 30 *μ*M ABA on *35S*:*TsRAVs* transgenic *Arabidopsis* seedling root elongation. Seedlings were grown on normal media for 5 days before being transferred onto 1/2 MS medium with 30 *μ*M ABA and grown for other 6 days. Each data bar represents the mean ± SE of three replicates. More than 50 seedlings were measured in each replicate. Different letters indicate significant differences among means (*P*<0.05 by Tukey’s test).

Abscisic acid (ABA) is a key phytohormone regulating many important plant growth and developmental events [39,43]. In the developing embryo, ABA accumulation regulates seed development, storage product accumulation, seed maturation, and seed dormancy [39,44]. The ABA content follows a rapid decline during imbibition process [45,46] and exogenous ABA inhibits seed germination and early seedling growth [38,39]. In the available literature, contrary results existed about the effect of *RAV* overexpression on the sensitivity of transgenic plant towards ABA inhibition during seed germination [4]. In this work, with the presence of 1 *μ*M ABA, seed germination was significantly inhibited in WT and all transgenic lines (Fig 5A). With the germination rate at DAG 3 of WT seeds changed from 100% to 46%, the average germination rate of *A-TsRAVs* and *B-TsRAVs* transgenic seeds dropped from 64% to 10% and from 81% to 38%, respectively (Fig 5A). When the inhibitory effects of ABA were calculated against their normal germination rates, *B-TsRAVs* transgenic seeds were affected to a same extent as WT seeds, but to a much less extent than *A-TsRAVs* transgenic seeds (Fig 5B). Our work was consistent with the previous report that exogenous ABA inhibited the germination of both wild type and *GhRAV1* overexpression transgenic seeds with a much greater degree of inhibition observed in the transgenic seeds than in wild type [18]. However, results contrary to our data also existed and a recent work revealed that *RAV1*-overexpressing lines showed strong ABA-insensitive phenotypes during seed germination and early seedling development, whereas *RAV1*-underexpressing lines were more sensitive to ABA than wild-type plants [15].

Since ABA inhibits the growth of *Arabidopsis* seedlings, we also investigated the ABA sensitivity of various transgenic lines in terms of seedling root growth. After transferring onto vertical agar plates supplemented with 30 *μ*M ABA, compared to the root growth of their respective untreated controls, both wild-type and all *TsRAVs* transgenic lines was similarly inhibited by about 50% (S4 Fig and Fig 5C), implied that RAV transcription factors might not play a direct role in ABA signaling during early seedling development. A previous work showed that 10 *μ*M ABA could strongly inhibit the root growth of *CaRAV1* overexpressing transgenic plants, but did not affect that of wild-type plants [10]. However, in another work, 10-day-old seedlings of wild type, *rav* mutant and *RAV2-*, *RAV3*-overexpressing *Arabidopsis* plants showed a similar reduction in root growth when exposed to 1 *μ*M of ABA [4], which demonstrated a normal responsiveness to ABA in terms of root growth inhibition as described for *TsRAVs* in our work. Interestingly, in the same work, the root growth of the *AtRAV1*-overexpressing plants was not inhibited by ABA treatment despite of their shorter initial root length, so the authors hypothesized that only RAV1 may be involved with the regulation of ABA sensitivity in *Arabidopsis* [4]. In contrast, data in this work indicated that overexpression of all 8 *TsRAV* genes did not alter the responsiveness of transgenic plants towards ABA and an altered ABA-sensitivity only occurred to the *35S*:*A-TsRAVs* transgenic plants at the seed germination stage.

**Fig 6:**
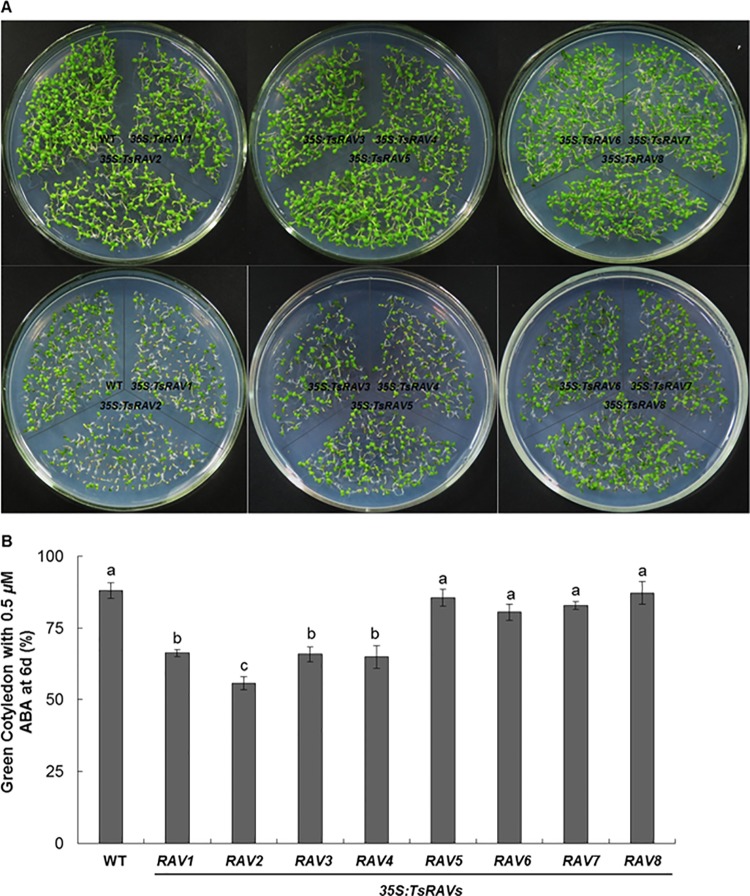
Cotyledon-greening analysis on *35S*:*TsRAVs* transgenic *Arabidopsis* seedlings. (A) Phenotypic comparison of wild-type and *35S*:*TsRAVs* transgenic *Arabidopsis* seedlings after grown on normal 1/2 MS medium (upper panel) or on 1/2 MS medium with 0.5 *μ*M ABA for 6 days (lower panel). (B) Cotyledon-greening percentages of *35S*:*TsRAVs* transgenic *Arabidopsis* seedlings after grown on 1/2 MS medium with 0.5 *μ*M ABA for 6 days. Each data bar represents the mean ± SE of three replicates. More than 100 seeds were measured in each replicate. Different letters indicate significant differences among means (*P*<0.05 by Tukey’s test).

It has been reported that when grown on MS plus ABA, the *RAV1*-overexpressing plants showed higher cotyledon-greening percentage than wild-type plants, and much lower cotyledon-greening percentages have been reported for *RAV* RNAi lines and *rav1* mutants [15], so we also determined the green cotyledon percentages of both wild type and *TsRAVs* transgenic lines in the current study. As shown in Fig 6A, there was no significant difference in green cotyledon rate between *TsRAVs* transgenic lines and wild type on normal 1/2 MS media, just as described previously [15]. In the presence of exogenous ABA, the green seedling rates of both wild type and *TsRAVs* overexpressing lines were significantly decreased. Compared to WT and *B-TsRAVs* transgenic lines, *A-TsRAVs* transgenic lines had significantly fewer open and green leaves after 6 days (Fig 6A). After being treated by 0.5 *μ*M ABA for 6 days, nearly 88% of wild type seedlings stayed green, while only 55–66% and 79–87% green seedlings was recorded for *A-TsRAVs* and *B-TsRAVs* transgenic lines, respectively (Fig 6B). Thus, the ABA-induced earlier leaf etiolation and lower chlorophyll content were much more severe in *35S*:*A-TsRAVs* transgenic lines, suggesting that *A-TsRAVs* but not *B-TsRAVs* might be involved in the ABA-regulated photosynthesis and leaf senescence.

### Water deficit tolerance of transgenic *Arabidopsis* overexpressing *TsRAVs*

**Fig 7:**
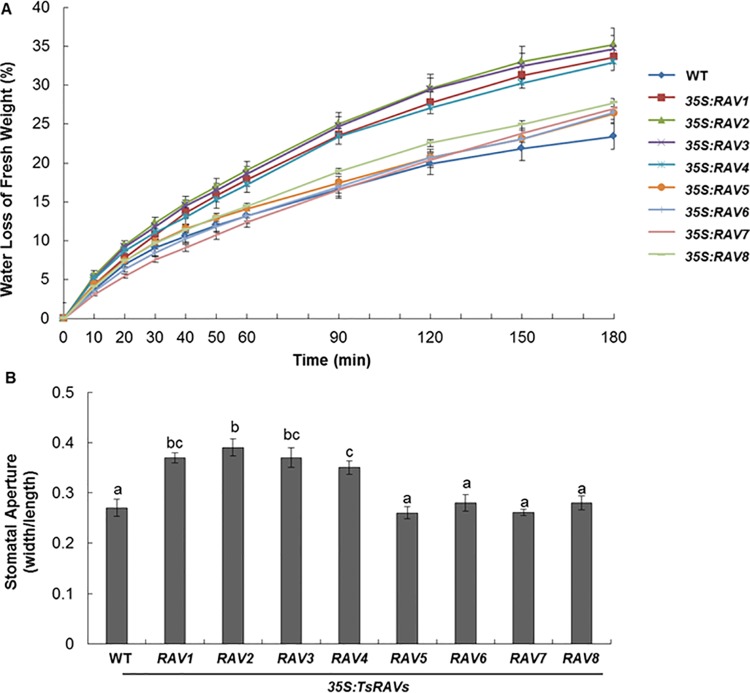
Different water loss rates of wild-type and *35S*:*TsRAVs* transgenic *Arabidopsis* plants. (A) Weight loss in fresh leaves under water deficit of 4-week-old wild-type and *35S*:*TsRAVs* transgenic *Arabidopsis* plants. Each data bar represents the mean ± SE of three replicates. (B) Width/length ratio of the rosette leaf stomatal aperture of 4-week-old wild-type and *35S*:*TsRAVs* transgenic *Arabidopsis* plants. Each data bar represents the mean ± SE of three replicates and different letters indicate significant differences among means (*P*<0.05 by Tukey’s test).

Water stress can induce the synthesis of ABA within plants and ABA acts directly on guard cells to induce stomatal closure and minimize water loss through transpiration [47]. Previously, transgenic plants overexpressing pepper *RAV1* have been shown to be more tolerant to water stress [10], whereas *AtRAVs*-overexpressing transgenic plants have been reported to exhibit higher transpirational water loss than wild type [4]. Thus, we cut leaves from the 4-week soil-grown *35S*:*TsRAVs* transgenic plants, placed them on a filter paper in an ambient temperature and measured the transpirational water loss over a 3-hour period. Our results showed that *35S*:*A-TsRAVs* experienced a significantly faster water loss than both wild type and *35S*:*B-TsRAVs* plants (Fig 7A). Since water loss of leaves is associated with the ABA-regulated stomatal movement, which is one of the appropriate criteria to check the ABA sensitivity, we then analyzed the stomatal opening of *TsRAVs*-overexpressing transgenic plants in parallel with wild type plants. Under same conditions, a wider opening of stomata was detected in *35S*:*A-TsRAVs* transgenic plants than both wild type and *35S*:*B-TsRAVs* transgenic plants (Fig 7B), indicating that constitutive overexpression of *A-TsRAVs*, but not *B-TsRAVs* could result in a wider stomatal opening, an increased transpiration rate, and a comprised drought tolerance, which was consistent with the previous notion that a faster water loss in *RAV1*-overexpressing plants was caused by the incapacity of stomata of to respond to ABA [4].

In summary, based on their distinctively different sequence characters and responses to salt and ABA stresses, RAV transcriptional factors originated from *Thellungiella salsuginea* could be divided into two major groups with four members within each group. Under normal conditions, *35S*:*A-TsRAVs* lines exhibited a moderately shortened root length, which indicated the roles of TsRAV1-4 as negative growth regulators. Base on the performance of each *TsRAVs* transgenic line under various ABA-related abiotic stresses, functional analyses revealed the highly consistent results among four members within a same group and distinctively different results between two groups. Compared to *35S*:*A-TsRAVs* plants, *35S*:*B-TsRAVs* plants only exerted a low degree or little inhibitory effect on seed germination and seedling growth, suggesting that ABA signaling pathway might have only been perturbed in *35S*:*A-TsRAVs* plants, but not in *35S*:*B-TsRAVs* plants. Moreover, the salt and ABA hypersensitivity conferred by *A-TsRAVs* overexpression only occurred to the germination stage, but not to the young seedling stage, indicating their inhibitory effect was modulated developmentally. Under water deficit conditions, *35S*:*A-TsRAVs* plants, but not *35S*:*B-TsRAVs* plants, became more susceptible to transpiration water loss than control plants due to their impaired stomatal aperture regulation. Therefore, despite of the presence of a B3 domain and a BRD repression domain in all eight TsRAVs, the presence or absence of a nuclear localization sequence, the core sequences within the BRD domains as well as some highly conserved group-specific residues might together affect their DNA-binding potentials and activities. Data present hereby suggests that two subsets of TsRAVs perform different physiological roles during plant growth, especially in ABA-mediated signaling pathway, and they might be regulated by different mechanisms and/or in turn modify different sets of downstream target genes.

## Supporting Information

S1 Fig Characterization of *35S*:*TsRAVs* transgenic *Arabidopsis* by semi qRT-PCR. Characterization of 35S:TsRAVs transgenic Arabidopsis by semi qRT-PCR. Four independent overexpression lines with similar level of transgene expression were chosen for the following analysis.
(TIF)

S2 Fig Phenotypes of representative line of *35S*:*TsRAVs* transgenic *Arabidopsis* plants grown in soil for 15 days. (TIF)

S3 Fig Germination rates of *35S*:*TsRAVs* transgenic *Arabidopsis* plants on 1/2 MS media with 50 mM or 75 mM NaCl. Germination rates of *35S*:*TsRAVs* transgenic *Arabidopsis* seeds on 1/2 MS media with 50 mM NaCl. Each data bar represents the means ± SE of three replicates. More than 100 seeds were measured in each replicate. Germination rates of *35S*:*TsRAVs* transgenic *Arabidopsis* seeds on 1/2 MS media with 75 mM NaCl. Each data bar represents the means ± SE of three replicates. More than 100 seeds were measured in each replicate.
(TIF)

S4 Fig Primary root length of *35S*:*TsRAVs* transgenic *Arabidopsis* seedlings grown on 1/2 MS media with 200 mM NaCl or 30 *μ*M ABA. After germination, seedlings were first grown on normal media for 5 days before being transferred onto 1/2 MS medium with 200 mM NaCl or 30 *μ*M ABA and grown for other 6 days.
(TIF)

S1 Table Gene-specific primers used in *TsRAVs* cDNA cloning and qRT-PCR analyses. (DOCX)

S2 Table Gene information of eight *Thellungiella salsuginea* and six *Arabidopsis thaliana RAV* genes. (DOCX)
