# Supplementary figures and images for: Two Groups of Thellungiella salsuginea RAVs Exhibit Distinct Responses and Sensitivity to Salt and ABA in Transgenic Arabidopsis
Source: PLoS One. 2016 Apr 19;11(4):e0153517. doi: 10.1371/journal.pone.0153517 (PMC4836749; doi:10.1371/journal.pone.0153517)

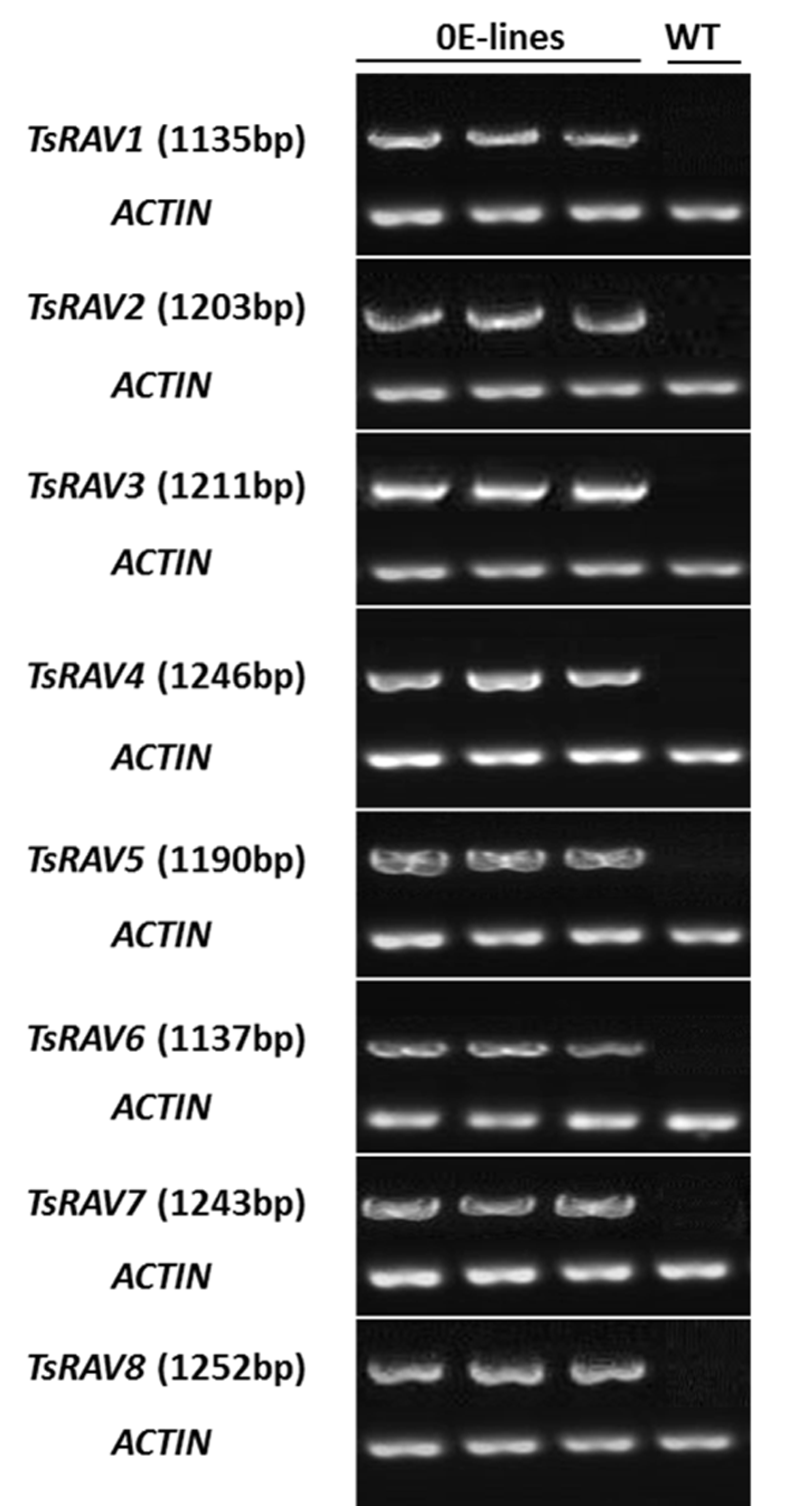

Supplement: S1 Fig — Characterization of 35S:TsRAVs transgenic Arabidopsis by semi qRT-PCR. Four independent overexpression lines with similar level of transgene expression were chosen for the following analysis. (TIF) [file pone.0153517.s001.tif]

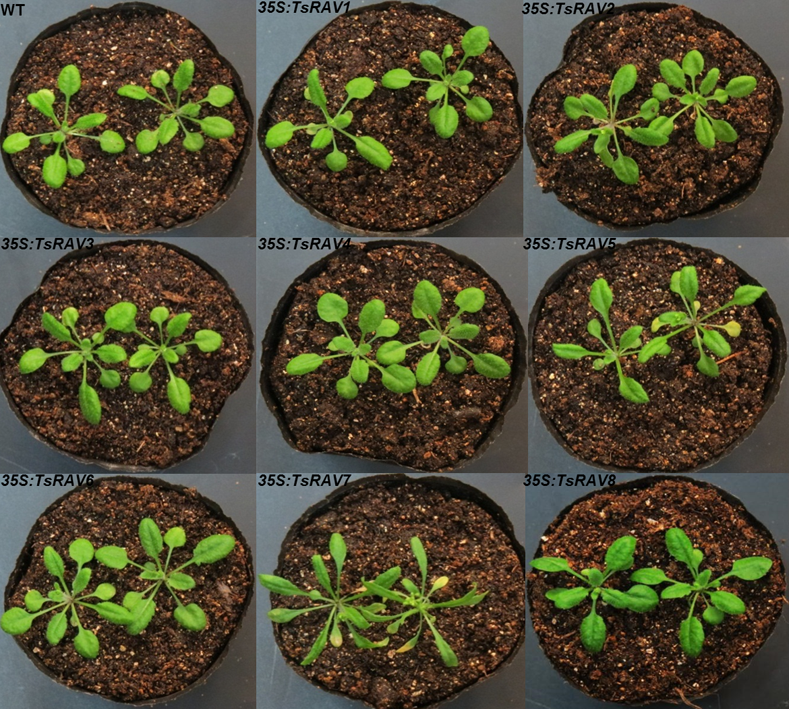

Supplement: S2 Fig — (TIF) [file pone.0153517.s002.tif]

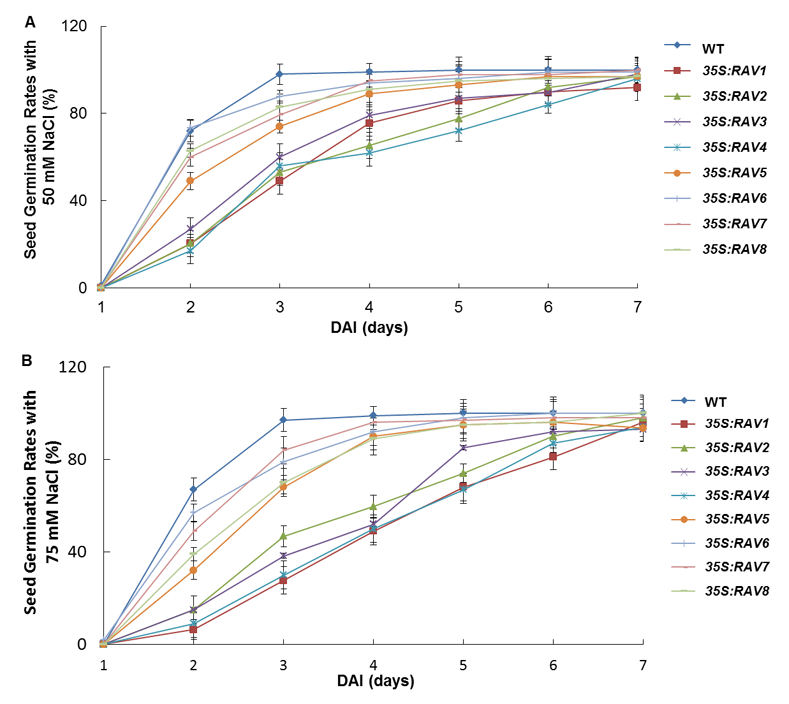

Supplement: S3 Fig — Germination rates of 35S:TsRAVs transgenic Arabidopsis seeds on 1/2 MS media with 50 mM NaCl. Each data bar represents the means ± SE of three replicates. More than 100 seeds were measured in each replicate. Germination rates of 35S:TsRAVs transgenic Arabidopsis seeds on 1/2 MS media with 75 mM NaCl. Each data bar represents the means ± SE of three replicates. More than 100 seeds were measured in each replicate. (TIF) [file pone.0153517.s003.tif]

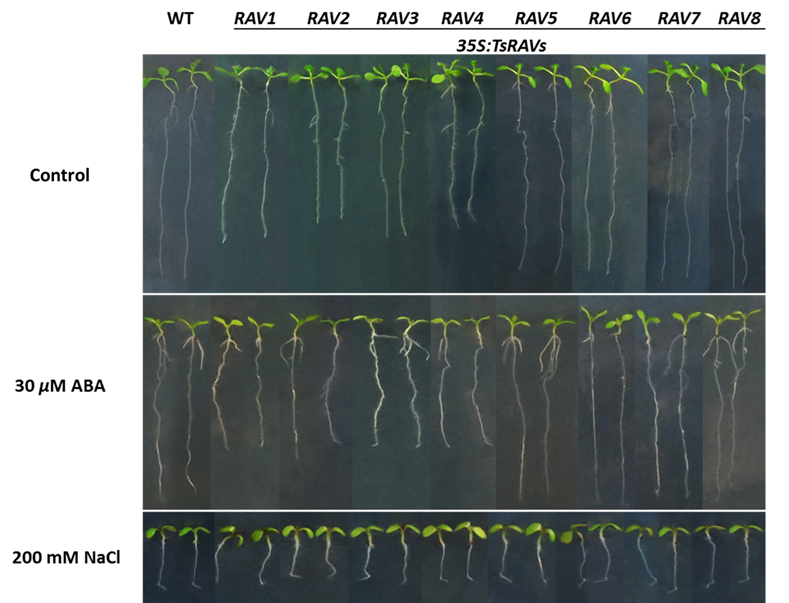

Supplement: S4 Fig — After germination, seedlings were first grown on normal media for 5 days before being transferred onto 1/2 MS medium with 200 mM NaCl or 30 μM ABA and grown for other 6 days. (TIF) [file pone.0153517.s004.tif]
