# Supplementary material for: Two Groups of Thellungiella salsuginea RAVs Exhibit Distinct Responses and Sensitivity to Salt and ABA in Transgenic Arabidopsis
Source: PLoS One. 2016 Apr 19;11(4):e0153517. doi: 10.1371/journal.pone.0153517 (PMC4836749; doi:10.1371/journal.pone.0153517)
Supplement: S1 Table — (DOCX) [file pone.0153517.s005.docx]

**S1 Table Gene-specific primers used in *TsRAVs* cDNA cloning and qRT-PCR analyses.**

| **Genes Name** | **Gene Locus** | **Primer Sequences for cDNA cloning** |
| --- | --- | --- |
| ***TsRAV1*** | ***Thhalv10010019m*** | F：ATACATTCCTGATTTCTCCGTTG  R： TATATCGAAGCAAAAAAAGGAGAG |
| ***TsRAV2*** | ***Thhalv10019566m*** | F：GTCACCCACATTTTCATCACC  R：GCGAAAATCACAGCCTACAACT |
| ***TsRAV3*** | ***Thhalv10004508m*** | F：CTCTCGATTCCCAAAATTTCAC  R：GGCAAGAAGAAGAAGTTAAAGACC |
| ***TsRAV4*** | ***Thhalv10007983m*** | F：CACAGCCCATTTCTCTTTCTCT  R：TGTCCTAATTTACAACTTGCCAAT |
| ***TsRAV5*** | ***Thhalv10012152m*** | F：TCTCTTCAGTGTTTTCTCAACCC  R：GCTTAAAAAGTGTCGCAACTCA |
| ***TsRAV6*** | ***Thhalv10012161m*** | F：GTATCCTCTGTTATTCCGATGGA  R：GTTGCAATTCAAAAGACGACCT |
| ***TsRAV7*** | ***Thhalv10012356m*** | F：TTGCTTATTGGCCGTAAATCTC  R：CAAGTTTGTAAATCAAGAGGCTACG |
| ***TsRAV8*** | ***Thhalv10012377m*** | F： CAAGCGACTCTTTCTTGCCTCT  R：AGGCGCAAACTATACATGATGC |
|  |  |  |
| **Genes Name** | **Gene Locus** | **Primer Sequences for qRT-PCR** |
| ***TsRAV1*** | ***Thhalv10010019m*** | F：GGAAGCGGATCTAGCGTGGT  R：GGAAGCGGATCTAGCGTGGT |
| ***TsRAV2*** | ***Thhalv10019566m*** | F：GATATGTTTGCGTTACGATGTTCTA  R：GCGAAAATCACAGCCTACAACT |
| ***TsRAV3*** | ***Thhalv10004508m*** | F：TGTGTTGACTAAAGGTTGGAGCA  R：CGTATCCTGATCCGACCCG |
| ***TsRAV4*** | ***Thhalv10007983m*** | F：CGAGTCTGGTTAGGCACT  R：CGTCATCACCGTCGTTGTCA |
| ***TsRAV5*** | ***Thhalv10012152m*** | F：TTTCAGGCGACGAGGAAGTAA  R：TCCTCTGCTTTGGTTTCTTCAC |
| ***TsRAV6*** | ***Thhalv10012161m*** | F：GTCCGAGAGCAAAAGCAACTCT  R：GATTTGAAAGTGCCGAGCCA |
| ***TsRAV7*** | ***Thhalv10012356m*** | F：GACAGGAACTGAAACCAAAACG  R：ATCTGAGCCCCCCAATGAC |
| ***TsRAV8*** | ***Thhalv10012377m*** | F：TTTCAGACAATGGTTTCGTGGA  R：CACGCCAAACAGCATAAACC |
| ***TsACTIN*** | ***Thhalv10020906m*** | F：GCACAATCCAAAAGAGGTATTCTCACCT  R：GGAGCCTCGGTAAGAAGAACAGGG |
| ***AtACT2*** | ***At3g18780*** | F：TGAGCACGCTCTTCTTGCTTTCA  R：GGTGGTGGCATCCATCTTGTTACA |
