# Supplementary material for: Two Groups of Thellungiella salsuginea RAVs Exhibit Distinct Responses and Sensitivity to Salt and ABA in Transgenic Arabidopsis
Source: PLoS One. 2016 Apr 19;11(4):e0153517. doi: 10.1371/journal.pone.0153517 (PMC4836749; doi:10.1371/journal.pone.0153517)
Supplement: S2 Table — (DOCX) [file pone.0153517.s006.docx]

**S2 Table** Gene information of eight *Thellungiella salsuginea* and six *Arabidopsis thaliana RAV* genes.

| **Gene** | **Gene Locus** | **Protein Size**  **(aa)** | **Molecular Weight**  **(kDa)** | **Isoelectric**  **Point** |
| --- | --- | --- | --- | --- |
| *TsRAV1* | *Thhalv10010019m* | 340 | 38.2 | 9.12 |
| *TsRAV2* | *Thhalv10019566m* | 350 | 39.4 | 9.54 |
| *TsRAV3* | *Thhalv10004508m* | 345 | 38.9 | 9.35 |
| *TsRAV4* | *Thhalv10007983m* | 367 | 40.9 | 9.20 |
| *TsRAV5* | *Thhalv10012152m* | 354 | 40.8 | 8.15 |
| *TsRAV6* | *Thhalv10012161m* | 320 | 37.0 | 8.98 |
| *TsRAV7* | *Thhalv10012356m* | 360 | 40.7 | 7.69 |
| *TsRAV8* | *Thhalv10012377m* | 384 | 43.6 | 5.47 |
| *AtRAV1* | *At1g13260* | 344 | 38.6 | 9.39 |
| *AtRAV2* | *At1g68840* | 352 | 39.5 | 9.39 |
| *AtRAV3* | *At3g25730* | 333 | 37.8 | 9.21 |
| *AtRAV4* | *At1g25560* | 361 | 40.6 | 9.30 |
| *AtRAV5* | *At1g51120* | 352 | 40.3 | 6.77 |
| *AtRAV6* | *At1g50680* | 337 | 38.5 | 6.62 |
